# Theoretical and Experimental Studies on Inclusion Complexes of Pinostrobin and β-Cyclodextrins

**DOI:** 10.3390/scipharm86010005

**Published:** 2018-01-30

**Authors:** Jintawee Kicuntod, Kanyani Sangpheak, Monika Mueller, Peter Wolschann, Helmut Viernstein, Saeko Yanaka, Koichi Kato, Warinthorn Chavasiri, Piamsook Pongsawasdi, Nawee Kungwan, Thanyada Rungrotmongkol

**Affiliations:** 1 Structural and Computational Biology Research Group, Department of Biochemistry, Faculty of Science, Chulalongkorn University, Bangkok 10330, Thailand; jintawee.ki@gmail.com (J.K.); karl.peter.wolschann@univie.ac.at (P.W.); 2 Starch and Cyclodextrin Research Unit, Department of Biochemistry, Faculty of Science, Chulalongkorn University, Bangkok 10330, Thailand; Piamsook.P@chula.ac.th; 3 Program in Biotechnology, Faculty of Science, Chulalongkorn University, Bangkok 10330, Thailand; swaratchada@gmail.com; 4 Department of Pharmaceutical Technology and Biopharmaceutics, University of Vienna, 1090 Vienna, Austria; monika.mueller@univie.ac.at (M.M.); helmut.viernstein@univie.ac.at (H.V.); 5 Institute of Theoretical Chemistry, University of Vienna, 1090 Vienna, Austria; 6 Institute for Molecular Science and Okazaki Institute for Integrative Bioscience, National Institutes of Natural Science, 5-1 Higashiyama Myodaiji, Okazaki 444-8787, Japan; saeko-yanaka@ims.ac.jp (S.Y.); kkatonmr@ims.ac.jp (K.K.); 7 School of Physical Sciences, SOKENDAI (The Graduate University for Advanced Studies), 5-1 Higashiyama Myodaiji, Okazaki 444-8787, Japan; 8 Natural Products Research Unit, Department of Chemistry, Faculty of Science, Chulalongkorn University, Bangkok 10330, Thailand; warinthorn.c@chula.ac.th; 9 Department of Chemistry, Faculty of Science, Chiang Mai University, Chiang Mai 50200, Thailand; naweekung@gmail.com; 10 Center of Excellence in Materials Science and Technology, Chiang Mai University, Chiang Mai 50200, Thailand; 11 Program in Bioinformatics and Computational Biology, Faculty of Science, Chulalongkorn University, Bangkok 10330, Thailand; 12 Molecular Sensory Science Center, Faculty of Science, Chulalongkorn University, Bangkok 10330, Thailand

**Keywords:** pinostrobin, β-cyclodextrin, inclusion complexation, biological activity, steered molecular dynamics simulation

## Abstract

Pinostrobin (PNS) belongs to the flavanone subclass of flavonoids which shows several biological activities such as anti-inflammatory, anti-cancerogenic, anti-viral and anti-oxidative effects. Similar to other flavonoids, PNS has a quite low water solubility. The purpose of this work is to improve the solubility and the biological activities of PNS by forming inclusion complexes with β-cyclodextrin (βCD) and its derivatives, heptakis-(2,6-di-*O*-methyl)-β-cyclodextrin (2,6-DMβCD) and (2-hydroxypropyl)-β-cyclodextrin (HPβCD). The A_L_-type diagram of the phase solubility studies of PNS exhibited the formed inclusion complexes with the 1:1 molar ratio. Inclusion complexes were prepared by the freeze-drying method and were characterized by differential scanning calorimetry (DSC). Two-dimensional nuclear magnetic resonance (2D-NMR) and steered molecular dynamics (SMD) simulation revealed two different binding modes of PNS, i.e., its phenyl- (*P*-PNS) and chromone- (*C*-PNS) rings preferably inserted into the cavity of βCD derivatives whilst only one orientation of PNS, where the *C*-PNS ring is inside the cavity, was detected in the case of the parental βCD. All PNS/βCDs complexes had a higher dissolution rate than free PNS. Both PNS and its complexes significantly exerted a lowering effect on the IL-6 secretion in LPS-stimulated macrophages and showed a moderate cytotoxic effect against MCF-7 and HeLa cancer cell lines in vitro.

## 1. Introduction

**Figure 1:**
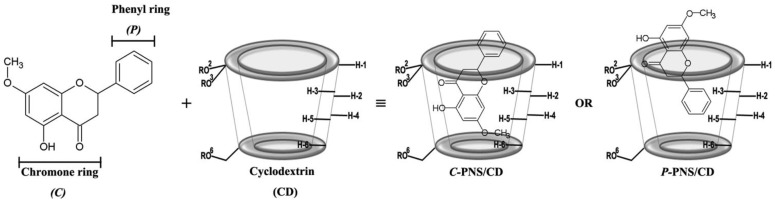
Two dimensional structures of two possible binding modes for the inclusion complex between pinostrobin (PNS) and cyclodextrin (CD) (shown as a truncated shape), where –R is –H, –CH_3_ and –C_3_H_7_O for βCD, 2,6-DMβCD (heptakis-(2,6-di-*O*-methyl)-β-cyclodextrin) and HPβCD ((2-hydroxypropyl)-β-cyclodextrin), respectively.

Plants are frequently used in the pharmaceutical fields, as functional foods or dietary supplements, since some of them contain various secondary metabolites including flavonoids, terpenoids, alkaloids, tannins, etc. [1] As shown in in vitro and in vivo studies, these metabolites have many biological activities such as cytotoxicity to cancer cell lines, anti-oxidative, anti-inflammatory as well as anti-microbial activities [2,3]. Pinostrobin (PNS, shown in Figure 1) or 5-hydroxy-7-methoxyflavanone is a secondary metabolite belonging to the flavanone subclass of flavonoids. These flavanones can be extracted from the rhizomes of fingerroot (*Boesenbergia rotrunda*) and galangal (*Alpinia galangal* and *A. officinarum*) as well as many other herbal plants mostly found in Southeast Asia. PNS has several important biological activities, such as an anti-viral role with the herpes simplex virus-1 [4], anti-mutagenic activity [5], anti-fungal effects for *Cytospora persoonii* [6] and anti-malarial activity [7]. It plays a major role in anti-ulcer activity that could be indirectly a result of its anti-oxidant mechanism [8]. From Wu and co-worker study [9], the results of in vitro 2,2-diphenyl-1-picrylhydrazyl (DPPH) scavenging assay also suggested that PNS shows anti-oxidant activity. Furthermore, PNS inhibits inflammatory cytokines, TNF-*α* (IC_50_ < 22 μM) and IL-1*β* (IC_50_ < 40 μM) in Sprague Dawley rats [10]. It has an effect on inhibiting aromatase activity and decreases the growth of MCF-7 cells (human breast cancer cells) induced by the dehydroepiandrosterone sulfate (DHEAS) and the estrogen receptor of 17*β*-estradiol (E_2_) [11]. Cao and co-worker revealed that PNS exerts cytotoxicity against HeLa and HepG2 cell lines [12]. The toxicity of PNS was evaluated on Wistar rats and was found to give a LD_50_ > 500 mg/kg, corresponding to PNS being non-toxic and non-genotoxic [13]. Similar to most flavonoids, PNS shows relatively low water solubility that may lead to a limited usage in pharmaceutical and industrial applications. 

In recent studies [14,15,16,17], the enhancement of poor water solubility and the dissolution of herbal drugs is one of the main challenges in the pharmaceutical field. Experimental studies on phase solubility, dissolution, characterization and biological activities of inclusion complexes between insoluble compounds and various cyclodextrins (CDs) have been widely reported. Interestingly, physical and biological properties were notably improved by a complexation with modified βCDs rather than parental βCD. In these studies the favorable orientation of the guest molecule binding within the lipophilic cavity of βCDs was characterized with various techniques e.g., differential scanning calorimetry (DSC), nuclear magnetic resonance (NMR) spectroscopy [18,19,20] and X-ray crystallography [21,22], as well as computational simulations [23,24,25]. For example, the solubility and anti-oxidant activity of apigenin was significantly increased with a 1:1 molar ratio between several βCD derivatives and apigenin [14]. The geometry of albendazole, a drug used in the treatment of gastrointestinal helminthic infections was characterized by DSC and two-dimensional NMR spectroscopies with a 1:1 ratio of albendazole with βCDs (βCD, hydroxypropyl-βCD, methyl-βCD and synthesized βCD-citrate). The inclusion complex of albendazole with βCD derivatives showed a higher solubility and dissolution [15]. The asymmetric free energy profiles resulting from the steered molecular dynamics (SMD) simulations of the three guest molecules, i.e., puerarin, daidzein and nabumetone, binding to βCD suggested two different types of inclusion complexes, B-ring of guest molecule inserting into βCD cavity from the primary rim (BP) and from the secondary rim (BS) [16]. With respects to solubility enhancement of PNS, by complexation with cyclodextrins in 1:1 molar ratio, studies have been performed using the suitable βCD derivative, heptakis-(2,6-di-*O*-methyl)-β-cyclodextrin (2,6-DMβCD). These studies were carried out using computational techniques that were experimentally validated by a phase solubility study at 25 °C in comparison to the parental βCD and other derivatives [17]. Herein, we further investigate the effect of temperature on the solubility of PNS in complex with βCD, 2,6-DMβCD and HPβCD with phase solubility studies. DSC and 2D-ROESY (Two-dimensional rotating-frame overhauser effect spectroscopy) investigations were carried out to characterize the solid inclusion complexes. Furthermore, the dissolution and biological activities of inclusion complexes were examined relative to PNS alone. The preferable binding mode of PNS inside the CD cavity was studied with a SMD approach. 

## 2. Materials and Methods

PNS was extracted from the fingerroot rhizome (*Boesenbergia rotrunda*) [26]. βCD and its derivatives (heptakis-(2,6-di-*O*-methyl)-β-cyclodextrin (2,6-DMβCD) and (2-hydroxypropyl)-β-cyclodextrin (HPβCD)), in technical grade, were purchased from Wako pure chemical industries, Ltd., and Sigma Aldrich, respectively. Methanol, in HPLC (High-performance liquid chromatography) grade (99.8% purity), was purchased from Wako pure chemical industries, Ltd. Deuterium oxide (D_2_O) and dimethyl sulfoxide-*d*_6_ (DMSO-*d*_6_) were obtained from Cambridge Isotope Laboratories, Inc. (Tewkesbury, MA, USA), and Euriso-top, respectively. Sodium dodecyl sulfate (SDS), thiazolyl blue tetrazolium bromide (MTT), compounds for phosphate buffer saline (PBS) and Lipopolysaccharides (LPS) from *Escherichia coli* O111:B4 were purchased from Sigma-Aldrich (Darmstadt, Germany). Dulbecco’s Modified Eagle’s medium (DMEM) and anti-mouse IL-6 were obtained from Life Technologies, Carlsbad, CA, USA and eBioscience Inc., San Diego, CA, USA, respectively. The murine macrophage cell line (RAW 264.7), human cervical carcinoma (HeLa) cells and breast cancer (MCF-7) cells, were obtained from the American Type Cell Culture Collection (ATCC), Manassas, VA, USA.

### 2.1. Phase Solubility Study

An excess amount of PNS was dissolved in pure water and in increasing amounts (0–10 mM) of βCD, 2,6-DMβCD and HPβCD, respectively. The mixtures were shaken in a water bath at 25, 30, 37 and 45 °C, for 72 h. The dissolved PNS concentration at each temperature was determined by UV-Vis spectrophotometry at 290 nm, which is the maximum absorption wavelength of PNS. Each experiment was performed in triplicate. Furthermore, the solubility of PNS in deionized water was determined in the same manner. The phase solubility diagrams of PNS in the presence of various concentrations of βCD and its derivatives were obtained using the same method as Higuchi and Connors [27]. The stability constants ($K_{C}$) of PNS when dissolved in different βCDs at various temperatures were calculated from the slope of the phase solubility linear diagrams according to Equation (1):

(1) $$K_{C}=\frac{Slope}{S_{0}\left(1-Slope\right)}$$

where $S_{0}$ is the saturation concentration of PNS in water at each temperature.

### 2.2. Preparation of Solid Inclusion Complexes

The solid inclusion complex between PNS and each cyclodextrin was prepared with the physical mixing and the freeze-drying methods, which resulted in a 1:1 molar ratio of PNS and CD. 

Physical mixing method: PNS, βCD and its derivatives were accurately weighed with equivalent amounts. Afterwards, PNS and each CD were physically mixed at room temperature. A portion of the resulting mixtures were used as controls and kept in a desiccator for further analysis.

Freeze drying method: PNS and the CDs were accurately weighed, and dissolved in 30-mL of deionized water. The solutions were stirred with a magnetic stirrer until the compounds were completely dissolved. The solutions were fully frozen at −80 °C overnight and subjecting to the lyophilizer (LYO-LAB, Lyophilization Systems, Telangana, India) for 2 days. The freeze-dried powder products were kept in a desiccator for further analysis.

### 2.3. Characterization of Inclusion Complexes

Differential Scanning Calorimetric analysis (DSC): DSC thermograms were obtained using the DSC 822e apparatus (Mettler Toledo, Columbus, OH, USA). All solid complexes were characterized using 0.7–2.0 mg of samples placed in aluminium pans and heated at a rate of 10 °C/min from 50 to 300 °C.

Two-dimensional Nuclear Magnetic Resonance (ROESY-NMR) spectra: The three solid inclusion complexes (PNS/βCD, PNS/2,6-DMβCD and PNS/HPβCD) were dissolved in suitable solvents. PNS/2,6-DMβCD and PNS/HPβCD were dissolved in 99.8% D_2_O whilst the less soluble PNS/βCD complex was dissolved in 1:3 DMSO-*d*_6_:D_2_O. Two-dimensional ROESY spectra of all inclusion complexes were obtained by using the AVANCE-500 spectrometer equipped with a 5 mm triple-resonance cryogenic probe (Bruker Biospin, Osaka, Japan). The NMR data were processed and analyzed using TopSpin (Bruker BioSpin) software [28,29]. The mixing time was set to 500 ms or 700 ms. The assignments of the pinostrobin and β-cyclodextrin proton signals can be found in the references [30,31,32].

Analysis of dissolution diagram: Equivalent amounts of free compound (PNS) and freeze-dried complexes were accurately weighed, and dissolved in 20-mL of Milli Q water. Then the solutions were shaken at 37 °C, 100 rpm for 4 h. At the same time, 1 mL of each solution was withdrawn at different time intervals (0, 5, 10, 15, 20, 30, 40, 60, 120, 180 and 240 min). Each sample was filtrated with 0.45 μm Nylon filter. A C-18 HPLC column was used to analyze the concentrations of dissolved PNS at 290 nm with the mobile phase consisting of 70:30 MeOH:Milli Q water. 

### 2.4. Determination of Biological Activity

To prepare the 10^−1^ μM stock solutions, each of the free PNS compound and its three inclusion complexes (PNS/βCD, PNS/2,6-DMβCD and PNS/HPβCD) were dissolved in absolute ethanol and distilled water, respectively, for anti-inflammatory and cytotoxicity assays. 

Anti-inflammatory assay: The murine macrophage cell line, RAW 264.7, was used to investigate the anti-inflammatory effect of the free compound and its inclusion complexes as described in previous work [33,34]. Cells were cultivated in 24 wells of DMEM medium with a density of 2 × 10^6^ per well and incubated at 37 °C for 24 h. Samples were added to the cultured cells and pre-incubated for 3 h before adding lipopolysaccharide (LPS) to the final concentration of 1μg/mL. After incubation of the cells at 37 °C for 24 h, the supernatant was used for enzyme linked immuno sorbent assay (ELISA) to determine the concentration of secreted IL-6. The ELISA was examined at room temperature following the manufacturer’s procedure (eBiosciences, Santa Clara, CA, USA). The optical density was measured at 450 nm using an Infinite M200 microplate reader (Tecan, Crailsheim, Germany), which was corrected with the reference wavelength of 570 nm. The cells that were attached to the bottom of the wells were incubated with MTT for 3 h at 37 °C. After that cells were lysed with a buffer containing 10% SDS in 0.01 N HCl and the produced formazan (by viable cells) was measured at 570 nm and corrected with the reference wavelength of 690 nm. Note that the untreated and treated LPS cells acted as the negative and positive controls, respectively. The positive control was defined as 100% cytokine secretion. All experiments were performed in triplicate. The half maximum inhibitory concentration (IC_50_) of the IL-6 secretion was determined using Table Curve 2D (Systat Software, San Jose, CA, USA).

Cytotoxicity assay: A MTT assay based on the conversion of MTT by mitochondrial dehydrogenase of viable cells to formazan was used to determine the cell viability of two different cancer cell lines (the cervix carcinoma cell line HeLa and the breast cancer cell line MCF-7) treated with free PNS and its inclusion complexes. This allowed the determination of the cytotoxicity of the samples against cancer cell lines. Briefly, cells were seeded into 96 wells with a density of 2 × 10^6^ per well and incubated at 37 °C for 24 h. The cells were subsequently incubated for a further 24 h with the compound solutions. Then cells were incubated with MTT (0.5 mg/mL) for 2 h. Afterwards, a lysis buffer was added to lyse the cells. The formazan was measured at 570 nm with a reference wavelength of 690 nm. The positive control was the cells only incubated with DMEM and defined as 100%. All experiments were performed in triplicate. The IC_50_ of the cytotoxic effect was determined using Table Curve 2D.

### 2.5. Computational Studies

#### Steered Molecular Dynamics Simulation

The docked structure of all inclusion complexes (PNS/βCDs) was obtained from a previous study [17] since the NMR results gave the structural information similar to our previous study. The Amber Glycam06 carbohydrate FF and its modified force field [35] and the GAFF parameters were applied to native βCD and its derivatives and PNS, respectively. These were then converted into a proper format for GROMACS by AnteChamber PYthon Parser interfacE (ACPYPE) [36]. All steered molecular dynamics simulations were performed with the GROMACS package (version 4.6.5). Each system contained an inclusion complex surrounded with approximately 3200 SPC water molecules in a cubic box of 4.5 × 4.5 × 4.5 nm^3^. PNS was then set at the center of the box with the Z-coordinate of cyclodextrin approximately located at Z equal to 2 nm. To equilibrate the simulated system prior to production, the whole system was minimized with 50,000 steps with the steepest descent algorithm (SD) and then 20,000 steps with the conjugated gradient algorithm (CG). Under periodic boundary condition with an *NPT* ensemble, the system was heated at a constant temperature of 289 K for 1 ns. The particle mesh Eward (PME) approach was used to treat long-range electrostatic interactions with non-bonded cut-off of 1 nm. The core CD was restrained and used as an immobile reference for ligand pulling. PNS was pulled out from the lipophilic cavity of CD along an axis perpendicular to the plane described by the secondary rim (wider rim) with a harmonic force constant of 2000 kJ mol^−1^ nm^−1^ and a pulling rate of 0.0010 nm ps^−1^ for approximately 2 ns. 

## 3. Results and Discussion

### 3.1. Phase Solubility Study

**Figure 2:**
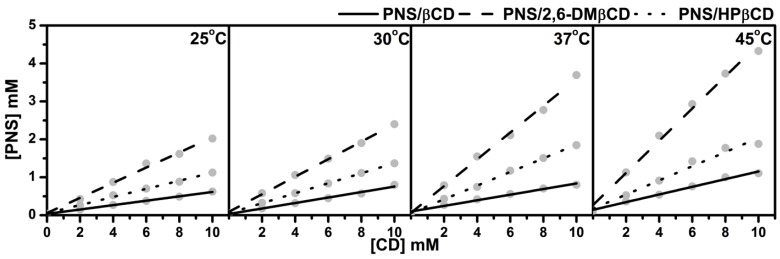
Phase solubility diagram for PNS with various concentrations of (—) βCD, (---) 2,6-DMβCD and (···) HPβCD at different temperatures.

The phase solubility diagrams of PNS/βCDs at temperatures 25, 30, 37 and 45 °C are plotted in Figure 2. The linear relationship showed A_L_-type phase solubility with 1:1 stoichiometry of PNS and CD inclusion complexes for all host molecules studied. The order of solubility at each temperature was ranked as PNS/2,6-DMβCD > PNS/HPβCD > PNS/βCD. A similar trend in phase solubility of inclusion complexes with βCDs has been reported in previous studies with various guest molecules [37,38]. 

Based on the method of Higuchi and Connors [27], the stability constants ($K_{C}$), of the inclusion complexes at the individual temperatures evaluated from the slope derived from Figure 2 and the intrinsic solubility of PNS ($S_{0}$) using Equation (1), are given in Table 1. Of note is that the stability constants calculated with this method are quite sensitive to the intrinsic solubility ($S_{0}$) because the intercept cannot be measured with good accuracy in cases where the solubility of the free compound is very low. Therefore, in the present study $S_{0}$ was determined directly as the PNS concentration in deionized water without any CD at each temperature. Table 1 shows the ranked $K_{C}$ values of PNS/2,6-DMβCD >> PNS/HPβCD > PNS/βCD for all temperatures. $K_{C}$ of PNS in complex with 2,6-DMβCD had a higher value than βCD and HPβCD with 4–6 folds and 2–3 folds, respectively. Since the stability constants seems to be responsible for binding behaviors between guest and host molecule, this data indicate that PNS is strongly interacting with the 2,6-DMβCD compared to βCD and HPβCD. The 2,6-DMβCD complex is more stable than the other complexes. Interestingly, an increase in temperature would reduce the stability of the PNS/βCD and PNS/HPβCD complexes, while no significant temperature effect was observed for the PNS/2,6-DMβCD complex. 

Using a Van’t Hoff plot [39], the thermodynamics parameters, enthalpy ($\Delta H^{\mp }$) and entropy ($\Delta S^{\mp }$), of the inclusion processes were obtained by plotting the stability constant ($K_{C}$) in Table 1 against the reciprocal temperature using Equation (2), while the Gibbs free energy at 25 °C ($\Delta G_{25}^{\mp }$) was evaluated using Equation (3). The thermodynamic parameters for all three PNS/βCDs are summarized in Table 2.

(2) $$\ln K_{C}=-\frac{\Delta H^{\mp }}{RT}+\frac{\Delta S^{\mp }}{R}$$

(3) $$\Delta G_{25}^{\mp }=\Delta H^{\mp }-T\Delta S^{\mp }$$

The negative enthalpy energies of −17.2 and −15.1 kJ/mol for PNS/βCD and PNS/HPβCD suggested that the complex formation is an enthalpy-driven exothermic process. This is in contrast for PNS/2,6-DMβCD complexation, where the small (non-significant) endothermic process was compensated by the entropy (a slightly positive enthalpy of 1.0 kJ/mol and a large positive entropy term of 22.9 kJ/mol). Such pronounced changes of the inclusion mechanisms were found also in other host-guest systems [40,41]. From this principally positive entropy value, it is possible that this complex might have a “loose” fit between PNS and the 2,6-DMβCD cavity, causing higher translational and rotational degrees of freedom for the 2,6-DMβCD and the structural breakdown of water molecule that surround the PNS molecule. From the Gibbs free binding energy, of the host and guest molecules, it can be concluded that all inclusion complexes are spontaneously formed with negative $\Delta G_{25}^{\mp }$ values. The order of the complex stabilities, or binding strengths, is as follows: PNS/2,6-DMβCD (−21.9 kJ/mol) > PNS/HPβCD (−20.5 kJ/mol) > PNS/βCD (−18.8 kJ/mol). 

### 3.2. Characterization of Inclusion Complexes

#### 3.2.1. Differential Scanning Calorimetriy

**Figure 3:**
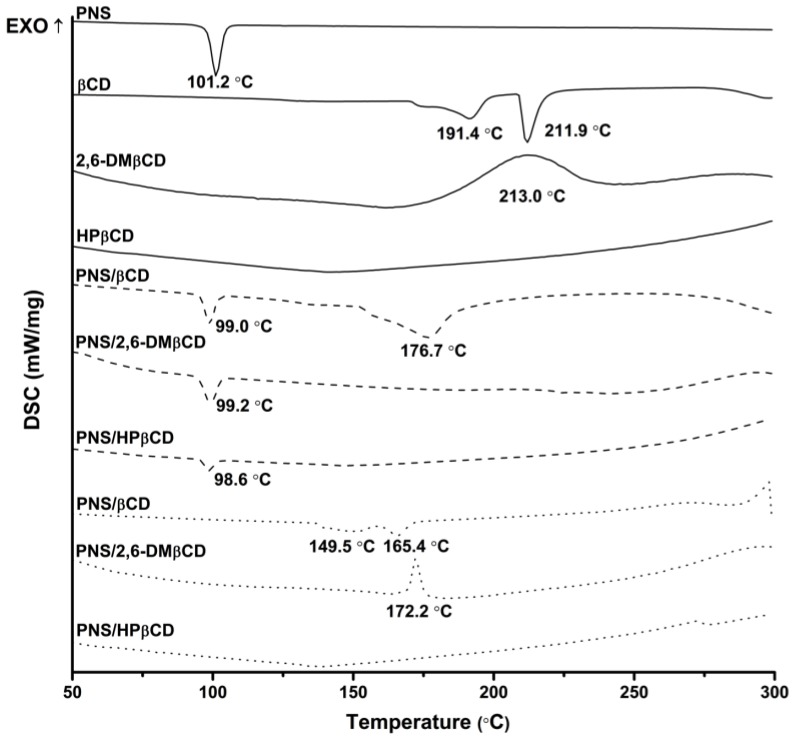
Differential scanning calorimetry (DSC) thermogram of free compounds (—) and PNS/βCDs inclusion complexes prepared by physical mixture (---) and freeze-drying method (···).

DSC was used to investigate the physicochemical and thermal behaviors of free PNS and each CD with their inclusion complexes. The resulting thermograms are shown in Figure 3. The sharp endothermic peak of free PNS at 101 °C refers to its melting point. The thermogram of free βCD present a prominent endothermic peak at 212 °C, while those of 2,6-DMβCD and HPβCD displayed a broad peak at approximately 213 °C and 134 °C, respectively. The distinct melting endotherm peak of free PNS was expressed at approximately 99 °C in the physical mixture products of all complexations. As can be seen from the superimposition in thermograms between the free compounds of PNS and βCDs, the physical mixture method seems not to give a real inclusion complex as observed elsewhere [42,43,44]. For freeze-dried products however, this endothermic peak of free PNS totally disappeared, while the thermal peaks of βCD and its derivatives were dramatically shifted with a decreased intensity. Therefore, the formation of a real inclusion complex between PNS and each CD could be obtained with the freeze-dried method owning to the replacement of water with PNS and the reduction of the maximum dehydration peak [45]. The freeze-dried method was already successfully applied to produce various inclusion complexes such as vanillin/βCD [43], quinestrol/2,6-DMβCD [46] and diclofenac/HPβCD [47]. 

#### 3.2.2. Two-Dimensional Nuclear Magnetic Resonance

**Figure 4:**
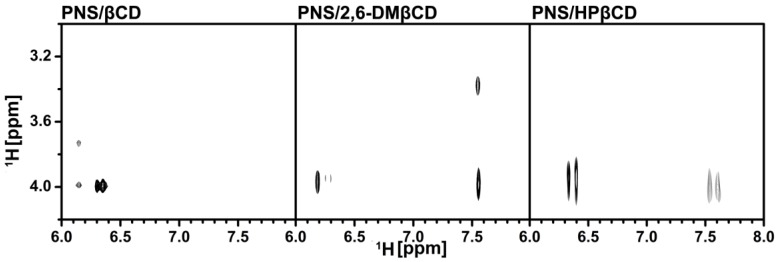
Two-dimensional ROESY spectra of the freeze-dried inclusion complexes.

To clarify the configuration of PNS in the hydrophobic cavity of βCDs, 2D-ROESY-NMR was used to examine the three freeze-dried inclusion complexes. A partial contour plot of proximities between the protons of PNS and βCDs is shown in Figure 4. From the PNS/βCD inclusion complex, the intermolecular cross-peak between the H-3 of βCD (~4 ppm) and the H-6 or the H-8 of PNS (6.2–6.4 ppm) is observed, which is a part of the chromone ring. The βCD derivative inclusion complexes had an additional correlation peak, which is the cross-peak between the H-3 of the βCD derivatives (~4 ppm) and the H-2′, H-3′ and H-4′ of PNS (7.5–7.7 ppm) and is a part of the phenyl ring. Interestingly, in the case of the 2,6-DMβCD inclusion complex, the intermolecular cross-peak between 2-OCH_3_ of 2,6-DMβCD (~3.4 ppm) and the phenyl ring (7.5–7.7 ppm) was also found. These results indicated that the parental βCD inclusion complex had one orientation where the chromone ring is inside the cavity. However, the βCD derivatives inclusion complexes had two orientations with PNS inside the cavity, with either the chromone- or the phenyl-ring inside the cavity. In addition, the phenyl ring of PNS might be strongly interacting with the 2,6-DMβCD since both the 2-OCH_3_ and the H-3 of 2,6-DMβCD were available for interaction. In the previous work, two different types of inclusion complexes between HPβCD and epigallocathechingallate were also formed in a similar manner [48]. 

#### 3.2.3. Steered Molecular Dynamics

**Figure 5:**
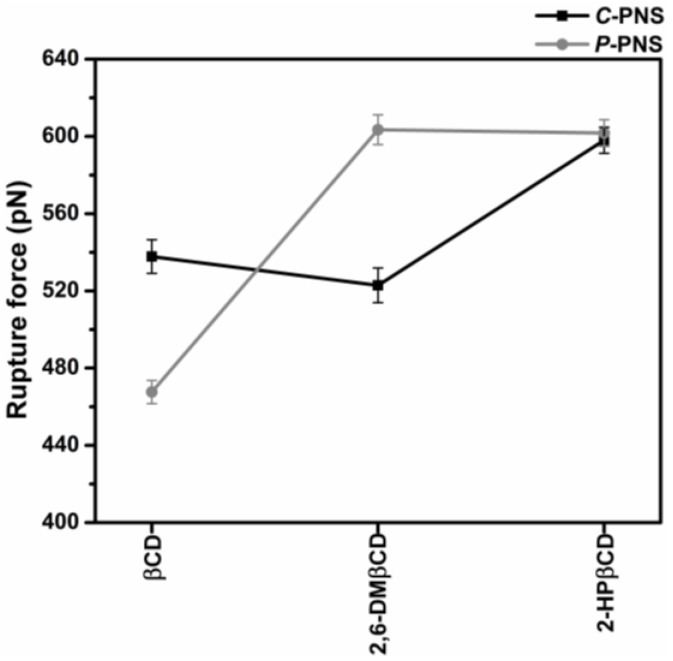
Rupture force (F_max_, pN) derived from the force-time profile of pulling the two oriented ligands, *C*-PNS and *P*-PNS, out from the wider rim along the host cavity axis for each inclusion complex.

Steered molecular dynamics (SMD) simulations are a flexible and powerful tool that is often used in scientific research in order to understand ligand-protein recognition, to investigate the dynamic behaviors of biological systems and also to assist in structure-based drug design and screening [49,50,51,52,53]. In this work, two possible orientations of PNS, *C*-PNS and *P*-PNS, encapsulated in the hydrophobic cavity of CDs in equilibrium state were separately pulled out by using an external force with identical spring constants. The force-time profile of the ligand being pulled out from the wider rim along the host cavity axis for each inclusion complex is depicted in Figure S1 in the Supplementary Materials. The highest rupture forces (F_max_) derived from Figure S1 for the six inclusion complexes with βCD, 2,6-DMβCD and 2-HPβCD are shown in Figure 5. Note that in this part 2-HPβCD was selected as the representative structure of the HPβCD mixture in accordance with the 2D ROESY NMR spectra on HPβCD, which showed that the degree of substitution (DS) was from 0.5 to 1 with regards to 2-hydroxypropyl substituted at the O2 position [54]. 

In the simulations of the complexes the pulling force reached the maximum value at approximately 500 ps, when the PNS molecule escaped from the nanocavity and the intermolecular interaction between PNS and βCDs (as described in our previous work [17]) was broken. The results were somewhat in correspondence with the SMD study on progesterone/βCD complex [55]. Based on the hypothesis that a higher pulling force corresponds to a more favorable binding mode of the guest molecule in the complex, *P*-PNS preferably interacted with 2,6-DMβCD compared to the interaction of the parental βCD, whilst both orientations of *P*-PNS and *C*-PNS were almost equally favorable when binding with 2-HPβCD. The F_max_ values in order of largest to smallest are as follows, 2,6-DMβCD (603 pN) ~ 2-HPβCD (601 pN) > βCD (468 pN). This is in agreement with the phase solubility study discussed earlier, where it was found that the stability of PNS was improved to a greater degree when forming an inclusion complex with the two modified βCDs compared to the parental βCD. These results suggest that differences in pulling force, and binding strength, are caused by the guest molecules orientation inside the host cavity [16]. 

#### 3.2.4. Dissolution Diagram

**Figure 6:**
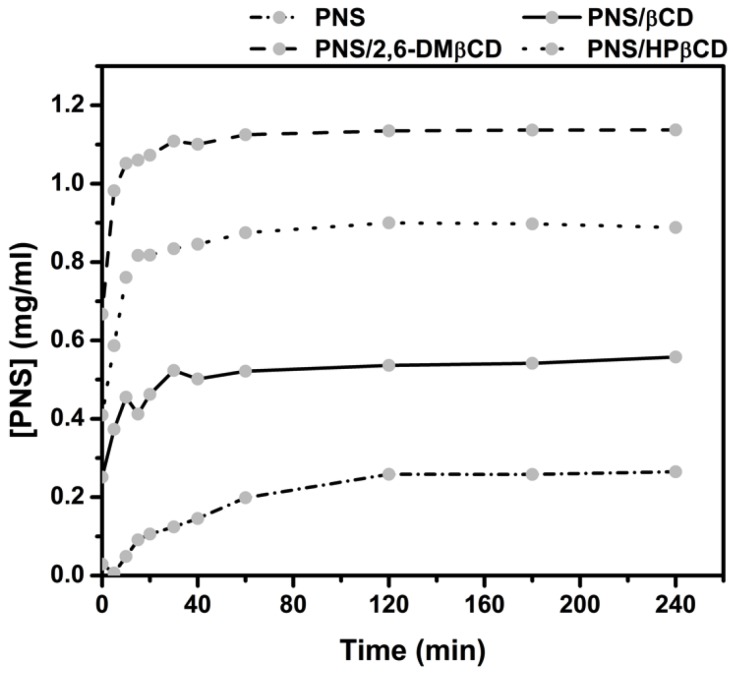
Dissolution diagrams of free PNS and its inclusion complexes with the three βCDs in water at 37 °C.

The in vitro dissolution studies of the freeze-dried complexes PNS/βCD, PNS/2,6-DMβCD and PNS/HPβCD were carried out in water at 37 °C for 240 min. The dissolution diagrams of these complexes relative to free PNS is depicted in Figure 6. It can be seen that free PNS had a rather poor dissolution in comparison with all inclusion complexes. These complexes showed a faster dissolution rate than free PNS in the initial phase. After 15 min, the amount of dissolved free PNS was lowered to only 0.09 mg/mL, while the dissolved amount of PNS complexed with βCD, 2,6-DMβCD and HPβCD were 0.41, 1.06 and 0.82 mg/mL, respectively. This is a 2-fold increase in the dissolution rate of the modified βCDs complexes compared to that of parental βCD. The reason for this could be that the –OCH_3_ group of 2,6-DMβCD has a greater interaction with the water molecules, whilst the hydroxypropyl moiety of HPβCD can form more hydrogen bonds and this may be reducing the aggregation behavior in water as previously reported in other work [56]. It has been suggested elsewhere that 2,6-DMβCD is the best carrier host among these three βCDs [57,58], where it is also suggested that the randomly methylated methyl-βCD can enhance the solubility of apigenin even more than HPβCD and the parental βCD [14]. 

#### 3.2.5. Determination of Inclusion Complexes Biological Activity

##### Anti-Inflammatory Activity

According to previous work, the effect of free PNS on nitric oxide, cyclooxygenase-1 and 2 induced by LPS/IFN-γ in RAW 264.7 cells is not significant [8]. In this work, the murine macrophage cell line (RAW 264.7) was stimulated by LPS and pre-treated with free PNS and three inclusion complexes (PNS/βCD, PNS/2,6-DMβCD and PNS/HPβCD). PNS and its complexes significantly exerted a lowering effect on the IL-6 secretion in LPS-stimulated macrophages. The IC_50_ values of free PNS (30 μM) and PNS/HPβCD (27 μM) (Table 3) are roughly comparable. For PNS/2,6-DMβCD and PNS/βCD the IC_50_ was slightly higher with 49 μM and 61 μM, respectively.

##### Cytotoxicity

The effect of PNS on many cancer cell lines has previously been reported elsewhere [59,60]. In particular, the leukaemia cell line, which is a neoplastic disease of the bone marrow and is also known as white blood cell cancer, was potentially inhibited by PNS [30,31]. However, the influence of PNS and its inclusion complexes on the cancer cell lines HeLa (cervical cancer cell line) and MCF-7 (breast cancer cell line) has not been widely reported until now. In this study, the cytotoxicity effect of PNS and its inclusion complexes (PNS/βCD, PNS/2,6-DMβCD and PNS/HPβCD) on these cancer cell lines was investigated. According to Table 3, the free compound and its complexes had a significant cytotoxic effect on the HeLa cell line with an IC_50_ value of 79 μM for the free PNS and IC_50_ values ranging between 65 and 95 μM for the CD complexes. Thus, the cytotoxic effect of the complexes is slightly, but not significantly different compared to the free PNS. Regarding the MCF-7 cell line, PNS/2,6-DMβCD is in some extent more cytotoxic than PNS with an IC_50_ value of 18 μM.

## 4. Conclusions

The solubility increase properties of the host-guest inclusion complexes of PNS with βCD and its derivatives were all of A_L_ type. It could be seen that PNS solubility increased with increasing concentration of βCDs. The highest solubility of PNS was exhibited in 2,6-DMβCD solution followed by HPβCD and then βCD solution at all examined temperatures. The endothermic peak of PNS in the DSC thermograms of the freeze-dried inclusion complexes completely disappeared, indicating that the freeze-drying method can prepare true inclusion complexes. Moreover, two-dimensional NMR (2D-ROESY-NMR) was employed to clarify the binding mode of PNS in the cavity of βCD and its derivatives. The NMR spectrum revealed that the *P*-PNS and *C*-PNS groups were located inside the βCD derivatives whereas only *C*-PNS was found to interact with the parental βCD. SMD simulation also showed that the parental βCD preferred to interact with the *C*-PNS more than *P*-PNS whilst the derivatives of βCD could interact with both of the *P*-PNS and *C*-PNS groups. According to the dissolution profile, the dissolution can be ordered as PNS/2,6-DMβCD > PNS/HPβCD > PNS/βCD > PNS. Free PNS and the PNS inclusion complexes lowered the secretion of the pro-inflammatory cytokine IL-6 in RAW 264.7 macrophages. Additionally, PNS and its inclusion complexes had a significant cytotoxic effect on MCF-7 as well as HeLa cancer cell lines. Of note is that the bioactivity of the free compound and the inclusion complexes was not significantly different.

## Figures and Tables

**Table 1:** Stability constant of all inclusion complexes at different temperatures.

Temperature (°C)	Stability Constant ($K_{C}$ in M^−1^)		
	PNS/βCD	PNS/2,6-DMβCD	PNS/HPβCD
25	1800	7320	3500
30	1580	6070	3070
37	1300	7560	2840
45	1190	6930	2370

**Table 2:** Thermodynamics values of the three inclusion complexes derived from Van’t Hoff plots (R: gas constant, T: absolute temperature).

Inclusion Complex	$\Delta H^{\mp }$ (kJ mol^−1^)	$T\Delta S^{\mp }$ (kJ mol^−1^)	$\Delta G_{25}^{\mp }$ (kJ mol^−1^)
PNS/βCD	−17.2	1.7	−18.8
PNS/2,6-DMβCD	1.0	22.9	−21.9
PNS/HPβCD	−15.1	5.4	−20.5

**Table 3:** The effect of pinostrobin and its complexes on the secretion of the pro-inflammatory cytokine IL-6 as well as cytotoxic effect towards HeLa and MCF-7 as indicated by their IC_50_ values.

	IC_50_ [μM]		
	IL-6 Secretion	Cytotoxicity Hela	Cytotoxicity MCF-7
PNS	30	79	22
PNS/βCD	61	72	28
PNS/2,6-DMβCD	49	95	18
PNS/HPβCD	27	65	29

## Supplementary Materials

The following are available online at http://www.mdpi.com/2218-0532/86/1/5/s1, Figure S1: The force-time profile of the ligand being pulled out from the wider rim along the host cavity axis for each inclusion complex.

Supplementary File 1
