# Supplementary material for: Theoretical and Experimental Studies on Inclusion Complexes of Pinostrobin and β-Cyclodextrins
Source: Sci Pharm. 2018 Jan 30;86(1):5. doi: 10.3390/scipharm86010005 (PMC5874535; doi:10.3390/scipharm86010005)
Supplement: Supplementary File 1 [file scipharm-86-00005-s001.pdf]

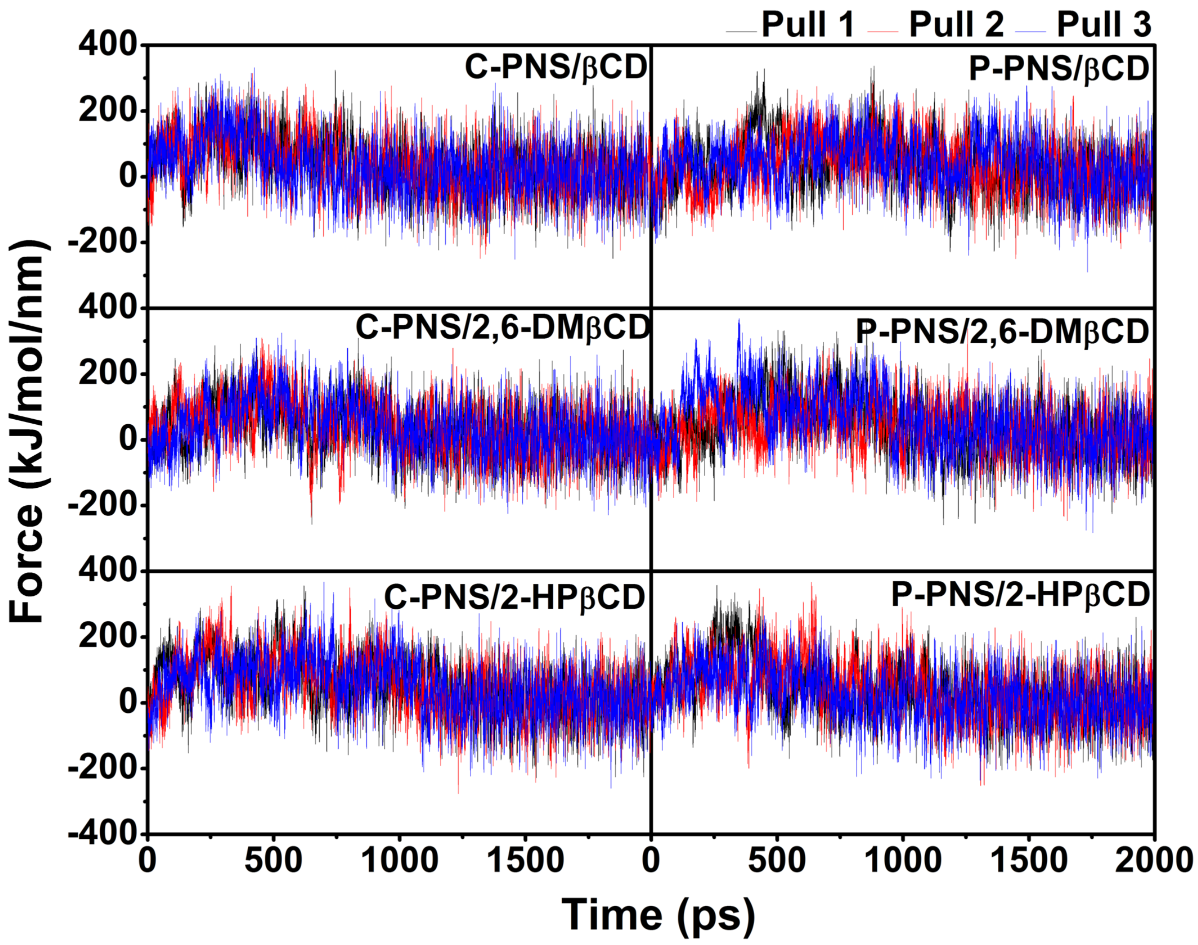

Fig. S1 The force-time profile of the ligand being pulled out from the wider rim along the host cavity axis for each inclusion complex
